# Migrants’ decision-process shaping work destination choice: the case of long-term care work in the United Kingdom and Norway

**DOI:** 10.1007/s10433-016-0405-0

**Published:** 2016-11-28

**Authors:** Karen Christensen, Shereen Hussein, Mohamed Ismail

**Affiliations:** 1 The Department of Sociology, Bergen University, Bergen, Norway; 2 The Policy Institute, King’s College London, Strand, London, WC2R 2LS UK; 3 Analytical Research Ltd, Station House, Surrey, GU24 0ER UK

**Keywords:** Labour market, Migration policy, The European Union, Social care

## Abstract

Escalating demands for formal long-term care (LTC) result in the reliance on migrant workers in many developed countries. Within Europe, this is currently framed by progressive European immigration policies favouring inter-European mobility. Using the UK and Norway as case studies, this article has two main aims: (1) to document changes in the contribution of European Union (EU) migrants to the LTC sectors in Western Europe, and (2) to gain further understanding of migrants’ decision-processes relating to destination and work choices. The UK and Norway provide examples of two European countries with different immigration histories, welfare regimes, labour market characteristics and cultural values, offering a rich comparison platform. The analysis utilizes national workforce datasets and data obtained from migrants working in the LTC sector in the UK and Norway (*n* = 248) and other stakeholders (*n* = 136). The analysis establishes a significant increase in the contribution of EU migrants (particularly from Eastern Europe) to the LTC sector in both the UK and Norway despite their different welfare regimes. The findings also highlight how migrant care workers develop rational decision-processes influenced by subjective perspectives of investments and returns within a context of wider structural migration barriers. The latter includes welfare and social care policies framing the conditions for migrants’ individual actions.

## Background

Like most European countries, the United Kingdom (UK) and Norway face serious demographic challenges due to ageing populations and simultaneous decline in the numbers and proportions of those of employment age (Andreassen 2010). These challenges escalate the demands for formal long-term care (LTC) that are anticipated to be higher than is possible to meet by projected growth in the countries’ labour markets (Skills for Care [SfC] 2012; Texmon and Stølen 2009). Challenges to recruitment to the sector include the complex way elderly care is financed and delivered (Simonazzi 2009; Vabø 2012); a discourse of deviance concerning older, ‘non-productive’, people in general, including ageism (Hagestad and Uhlenberg 2005) and the gendered, emotionally taxing, nature of work encompassing the construct of feminized care work as ‘bad jobs’ (England 2005).

This article aims to contribute to two current theoretical discussions in the literature of migration, ageing and care. One concerns the search for different models of demand for migrant workers according to different care regimes in European countries. In particular, we question Van Hooren’s conclusions (Van Hooren 2012)—using the cases of Italy, England and Netherlands—relating to how migrants are situated within the dimensions of care provision in relation to the country’s care model. Van Hooren finds that a familialistic care regime results in a ‘migrant in the family’ model, a liberal care regime results in a ‘migrant in the market’ model and a social democratic regime leads principally to no demand for migrant workers and is likely to attract native workers due to high public expenditures for services.

The second concerns the intersection between different institutional factors such as immigration policies, welfare policies and care regimes and their impact on the recruitment of migrant care workers (e.g. Da Roit and Weicht 2013; Van Hooren 2012). We incorporate in the analysis framework both *macro*-*social* structures (states’ immigration policies, welfare regimes and labour market dynamics) as well as the subjective dimension of migrants’ rational decision-processes. The latter is inspired by earlier work related to the concept of ‘migrant agency’, where agency refers to individual and group action, which helps people to cope in specific situations of change (Castles 2010). Within this framework, macro-social structures might have different meanings depending on the way they are interpreted and acted upon within a migrant agency, individual (or group) perspective. Using empirical data we examine how individual subjective considerations of investments and returns form an important aspect of destination and work choices in the context of increased demand for LTC jobs and current European free labour mobility. We draw on relevant literature related to factors impacting migratory destination choices such as access to welfare payments (Barrett and McCarthy 2008), expectations about educational opportunities (de Brauw and Giles 2008) and migratory networks in the host society (Hoang 2011).

### Migrants’ subjective decision process

One key element in the migration discussion relates to immigration policies steering the access to and between countries within political systems such as the European Union (EU) (Castles 2010). Linked to these structural conditions, is the bottom-up process through which migrants and their families actively shape the actual migratory decisions. While summarized in migration theory as ‘migrant agency’, in social action theory, more generally, this can be related to the rational choice theory (Barnes 2000; Coleman 1990). In essence, it argues that every individual weighs up perceived consequences of certain actions and then chooses those considered most beneficial in a specific situation. This theory emphasises the free (independent) choice and high degree of predictability of individuals (Barnes 2000, pp. 18–19). Our analysis focuses on migrants’ individual and subjective decision processes when considering moving to and working in the LTC sector^1^ in the UK or Norway based in part both on accurate and inaccurate assumptions made at the individual level but also shaped by structural conditions (Castles 2010; Coleman 1990; Williams 2012). Structural factors, including immigration legislation and welfare and labour market policies, determine the conditions under which migrants can enter, live and work in the host country, while the subjective process is the interpretation migrants apply through a migrant agency perspective. For example, a country might have tight immigration rules but it could be still perceived as accessible by individuals due to their own social networks and links or when migrants’ knowledge about skills’ shortages in care work is proactively used as a means of entering to a specific destination.

### European LTC sector reliance on migrant workers

Current research indicates that migrants comprise a considerable portion of the LTC workforce in both the UK and Norway. In both countries, along with the Netherlands and Sweden, migrants are often employed in the formal sector. This is unlike other countries such as Germany, Austria and Italy where migrant LTC workers are more concentrated in private households (Da Roit and Weicht 2013).

Traditionally, Europe has relied heavily on migrant care workers from outside Europe, especially from the Philippines and Africa to meet such needs (Ruhs and Anderson 2010). However, over the past decade, Europe has increasingly restricted labour mobility from outside the EU while facilitating inter-European mobility (Thorud 2010). Current literature highlights the importance of migrant workers in European care work in its different forms, including formal care, institutional as well as home-based elderly care and child care in private homes (Shutes and Chiatti 2012; Van Hooren 2012; Williams 2012). In the UK, migrant care workers constitute around 20% of the overall workforce and around 50% of workers in London and large cities (Skills for Care [SfC] 2012). They are on average younger and have higher qualifications than British workers, and also include a higher percentage of men (Hussein and Christensen 2016). In Norway, migrants represent 13% of the total number of work years in the sector (Ramm 2013). Compared to Norwegian workers, migrant care workers are younger, have less absence due to illness and include a higher percentage of men. They tend to have less relevant qualifications than their Norwegian counterparts (58 vs. 75%, respectively), though this might relate to difficulties qualifications obtained in their home countries being recognized (Ramm 2013). Current evidence suggests that migrant care workers in the UK and Norway have a similar profile in terms of age and gender compared to non-migrants working in the sector; however, migrants care workers in the UK appear to have higher qualification levels.

### The case studies of the UK and Norway

The choice of Norway and the UK as our case studies has conceptual importance in understanding individual European migrants’ decision process. While both countries are faced with similar challenges of securing, an adequate LTC workforce to meet growing demands associated with ageing populations, they differ in their demographic composition, immigration policy histories and welfare regimes. Thus, they offer a rich comparative platform allowing the investigation of structural and individual factors impacting on the migratory process of European workers who decide to, or end up, working in LTC in each of these countries.

The UK has relied extensively, for many decades, on immigration to fill labour shortages including the LTC sector, first, during the 1960s and 1970s from Commonwealth states, formerly part of the British Empire (Redfoot and Houser 2008). Following these immigration waves, the UK gradually began to closely link migration policies to economic imperatives such as redressing workforce shortages. The UK was one of a minorities of EU states that permitted free labour flows after A8 accession^2^ in 2004 (along with Ireland and Sweden; see Portes and French 2005). In 2008, the UK introduced a ‘point-based’ system, based mainly on the skills of individual migrants (Somerville 2009). This has reduced the ability of employers to recruit migrants from outside the EU into the LTC sector (Dobson and Salt 2009). More recently, in 2010, the UK has introduced an ‘immigration cap’ on non-EU migrants in order to further reduce the number of migrants from outside the EU.

The British LTC sector is funded through both public and private funds, with publicly funded social care provision accounting for only 20–25% of all people accessing services through a tight means-tested process (Baxter and Glendinning 2014). Private care provision is the norm with over three quarters of social care services provided by the independent sector (for-profit and non-profit) (Skills for Care [SfC] 2012). Overall, the UK LTC sector is characterized by poor pay and difficult working conditions and with fiscal cuts to local government LTC has become increasingly fragmented and casual (Rubery et al. 2015).

Norway allowed free access to the country until 1975 when the ‘immigration stop’ was implemented, although the ‘stop’ was merely an introduction of some form of immigration control (Brochmann and Hagelund 2010). Between 1975 and 2004, the largest groups of migrants came to Norway for humanitarian reasons or because they had family members living in Norway already. After the EU expansions, from 2004 to 2007, the profile of migrants entering Norway started to change with the fastest growth observed among economic migrants from East and Central Europe (Thorud 2010).

In Norway, LTC is funded through national and local taxation and services are in the main freely available (particularly home-based nursing services), but with some services based on limited income-based contributions. Since the millennium, the inclusion of market-based services has also gained policy interest, but with great local/municipal variations (Christensen 2012; Vabø et al. 2013). An implication of such a marketization process is a gradual change from a clear publicly financed and organized LTC to a mixed economy, including the option of public cooperation with private for-profit care providers (Vabø et al. 2013), known internationally for their interest in recruiting foreign workers (Cangiano et al. 2009).

## Data and methods

Using the cases of the UK and Norway, representing in Esping-Andersen’s differentiation an ‘increasingly liberal’ and a Social democratic welfare regime (Esping-Andersen 1999), we examine the patterns of reliance on migrant workers from central and Eastern Europe, using macro data on migrant LTC workers. We then compare the different decision-making processes of European migrants joining the UK and Norway LTC sectors.

To examine the first hypothesis that both the UK and Norway LTC sectors are increasingly relying on migrants from within Europe, we analysed national migration and labour data. For the UK, we used the National Minimum Data Set for Social Care (NMDS-SC), March 2014, which is the largest sector specific dataset and collects rich information on providers of LTC as well as the workforce. xxxEmployers identified the nationality of individual employees and the year they entered the UK; those identified in the dataset as not British nationals are being considered migrants in our analysis. While the dataset relates to England only, there is no other comparative data for the other countries of the UK (Scotland, Wales and Northern Ireland) and we do not expect the trends in migrant workers to be substantially different, given that migrants make the decision to move to the UK then subsequently work in any of the UK countries. We focus the analysis on changes occurring since 2003, the year before the UK allowed free labour mobility from the A8 accession countries.

For Norway, we used published national statistics downloaded from the Statistics Norway website (Statistics Norway 2014). The data provided aggregate numbers of employed migrants, identified as foreign born with parents and grandparents foreign born, according to year of entry to Norway, country of origin and type work of industry including the LTC sector since 2008. Countries of origin in the Norwegian dataset were grouped slightly differently from the grouping of the NMDS-SC from England. However, the grouping still provides good comparability as they both identify migrants from within and outside the EU. In the case of Norway, data are provided on the stock of migrants from different countries in the LTC sector by year. In an attempt to measure change and trends of migrants’ contribution to the LTC sector in relation to their country of origin, we examined the increased relative importance of different migrant groups to the sector since 2008, using data from 2008 as our reference point.

To address our second research, question on migrants’ subjective considerations influencing destination and work choices we utilize data derived from two studies the authors, respectively, have led focusing on LTC labour mobility in the UK and Norway (study A and B). Study A, took place from 2007 to 2010 and comprised interview data from different groups including migrant LTC workers, employers/managers, recruitment agencies, policy stakeholders, British care workers and service users. The study employed a stratified sampling approach, starting with six diverse local areas (multiple-study sites) in England employing a maximum-variation sampling technique (Maykut and Morehouse 2000). Participants were recruited through care providers, care agencies, flyers and local employment events. Interviews were conducted (Hussein and colleagues) using a semi-structured interview guide. Most of the interviews were conducted in English except for few in French and Arabic. Study A examined several topics related to the experience and contribution of migrant workers to the UK LTC sector. After initial analysis of interview data, an online national survey was designed and distributed to a national sample of other migrant care workers with the aim to test the generalizability of some of the findings identified in the six local areas. The call to the survey was distributed through social care providers, online forums for social care and migrant groups as well as the professional press. Specific to the current analysis, the survey attempted to examine key differences in the challenges faced by EU and non-EU migrant care workers (for details see Hussein et al. 2010, 2011, 2013; Hussein 2014).

Study B is a comparative Norwegian study carried out in 2011–2013. It comprised 51 life story interviews with respectively 20 and 31 migrant workers in the Norwegian and English LTC sector. The participants of the study were recruited using a purposive sampling approach (Stake 1994) due to the fact that migrant care workers appeared to be a hard-to-reach-group as many of them worked as ‘personal assistants’ in private households (Christensen and Guldvik 2014, p. 29). Participants were recruited through care providers as well as online advertising websites, and in Norway mainly through municipalities and non-profit as well as for-profit providers. While the UK arm of the study was carried out by Hussein in English, the Norwegian arm was carried out by Christensen in Norwegian. The aim of this study’s analysis was to compare migrant life course stories as cases of different intersections of biography and historical time (Elder 1994). Migrant care workers’ decision-processes therefore were included in the data.

Interviews and questionnaires of both study A and B collected information on the migratory journey of the workers, their choice of the country to migrate to and choice of sector to work in. The two studies collected detailed information on the role of social networks, availability of information on the destination country and immigration policies in their experiences of joining the UK or Norway. For full details of interviews and survey questions, see Hussein et al. (2010, 2011, 2013) and Christensen and Guldvik (2014). Table 1 presents key characteristics of migrant workers participating in studies A and B, and Table 2 presents an overview of interviews with other stakeholders from study A. Interviews were conducted in English or Norwegian, while the majority were able to complete the interviews comfortably in these languages, some—particularly migrants from Eastern Europe—participants in Norway had limited English or Norwegian language proficiency. The analysis presented here focuses on data collected from 80 EU migrants. Data from interviews with other stakeholders are used for comparative purposes or to stress key themes identified from interviews with EU migrants (see Table 2).

**Table 1:** Migrant care workers participating in studies A and B by main characteristics

Characteristics of migrants LTC workers	Study A: the UK		Study B: Norway/Britain	Total
	Interviews	Survey		
Gender				
Women	76	75	35	186
Men	20	26	16	62
Home country				
EU	23	35	22	80
A8^a^	13	10	14	37
A2^b^	1	3	3	7
Other EU	9	22	5	36
Non-EU	73	66	29	168
Mean age	36.5	37.4	36.8	36.9
Obtained LTC job before arrival	38	35	11	84
Obtained LTC job after arrival	58	66	40	164
Total number of participants	96	101	51	248

^a^A8 Accession countries who joined the EU in 2003: The Czech Republic, Estonia, Hungary, Latvia, Lithuania, Poland, Slovakia and Slovenia

^b^A2 refers to Romania and Bulgaria

**Table 2:** Additional interviews with various stakeholder, Study A, the UK

Stakeholder group	Number of interviews
Service users/carers	35
Human resource staff	12
Managers/employers	26
British LTC workers	28
Recruitment agencies	20
Policy stakeholders	15
Total	136

All interviews were recorded with permission and then transcribed. The interviews were thematically analysed, searching for decision-processes in the migratory journey, through a process of familiarization, themes’ identification, coding then refining by the authors (Gomm et al. 2000). It is worth noting that our sample of migrant workers includes only those who have already migrated from within Europe and were working at the time of data collection in the LTC sector in each of the two destination countries under study. While this may exclude other migrants who were not able to achieve either of these goals, we suggest that such exclusion serves a better understanding of the process involved in achieving both the migratory and LTC work outcome rather than examining unfulfilled goals.

## Results

### Increased reliance on European migrant workers in the Norwegian and British LTC sectors

**Fig. 1:**
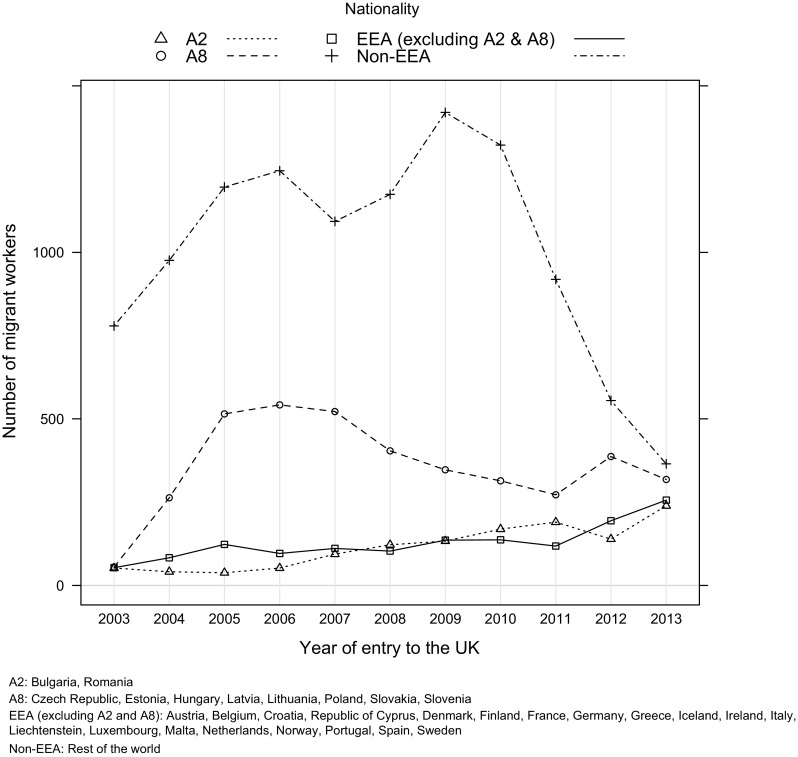
Trends of number of migrants working in the social care sector in England by year of entry to the UK and nationality, NMDS-SC 2014

Based on 543,572 care workers’ records obtained from the English NMDS-SC with nationality information, 18% were identified as migrants (not British nationals). The data, however, are likely to have underestimated the real contribution of migrants to the British LTC sector due to the fact that these records were completed by employers and it is possible that some migrants might be invisible to them, especially if they did not require work permits such as those arriving from within the EU. The vast majority of migrant workers in the UK LTC sector were from non-European Economic Area (EEA; see Fig. 1) countries (71%), with over a quarter of all migrants arriving from just two countries: the Philippines and India, though this profile has been quickly changing. Figure 1 shows the number of LTC migrant workers who have entered the UK from 2003 to 2013. The analysis shows a steady increase of migrant workers from within Europe especially those from A8 and A2 countries (Romania and Bulgaria who joined the EU in 2007), revealing that up until 2010, non-EU migrant LTC workers entering the UK continued to exceed those from the EU. From 2011 to 2013, this trend was reversed with much larger groups of LTC migrant workers from the EU entering the UK than other groups. For example, 813 European LTC (total of migrant workers from A2, A8 and other EEA countries) migrant workers were identified by employers to have entered the UK in 2013 compared to only 365 migrants from outside Europe. These trends are clearly linked to the progressive UK immigration policies of reducing migration from outside the EU with 2010 marking the introduction of the non-EU immigration cap discussed earlier.

**Fig. 2:**
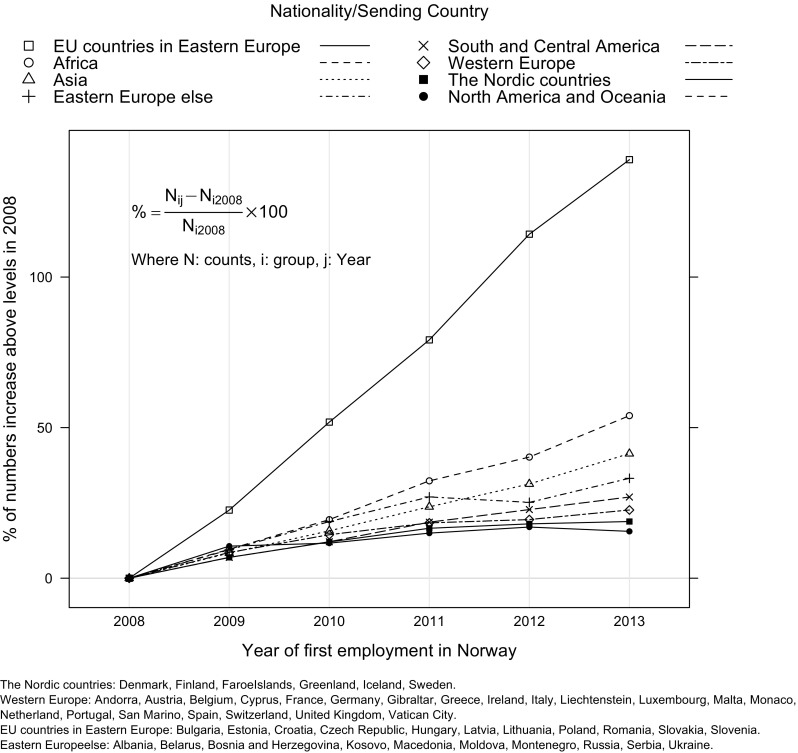
Trends in percentage increase in migrant LTC workers entering Norway above levels observed in 2008 by nationality, Statistics Norway

Figure 2 presents data from Norway showing that the largest growth since 2008 in LTC sector migrant-labour was among those arriving from EU countries in Eastern Europe. For example, compared to 2008, the number of workers from this group has increased by 139%. The number of migrants from other Eastern European countries that do not belong to the EU (such as Albania and Belarus) has also increased but not at the same rate. Data from both the UK and Norway confirm our hypothesis that the LTC sector in both countries is increasingly reliant on migrants from Europe, especially those from Eastern and Central Europe, in spite of their different welfare regimes and LTC sectors.

### From immigration policies to destination choices

Current immigration policies in both the UK and Norway share many similarities, particularly a direction favouring inter-European labour mobility. However, historical developments of these policies, and more importantly, the relationships of each of these countries with potential migrants and their lives are quite different. We found the perceived accessibility of the country and its association with migrants’ own individual personal history and life course to be important factors in migrants’ destination choices, even more than the actual immigration policy of the country (Castles 2010). Firstly, there was a positive historical relationship among many European migrants with the UK. For example, Antoni, a 32-year-old Polish man, considered coming to the UK because of his personal and family ties to the UK that are rooted in his family biography:

My father was doing Second World War in England and his brother was married in Scotland and was living in Ayr in Glasgow and brother of my mother was in [the] Polish army, was fighting in Africa, Italy Monte Casino, after Second World War living in London. I was many [times] in England, in Britain. I think that I know Britain sometimes better as English, because I was in many places. (Antoni, Poland, Study A)

In this sense, many European migrants in our study considered moving to the UK through capitalizing on their own family and social networks and previous knowledge to weigh up options, including post migration conditions as experienced by members of these networks. Jakub, a 29-year-old Polish man, explains how the idea of moving to the UK was initiated through his brother’s experience:

My brother, he was first in England and he call me. He tells me I can move to England. He asks me, do you want to come here and work in care? (Jakub, Poland, Study A)

The destination choice of Norway, on the other hand, appeared to be developed more indirectly, still based on an active choice, but considered through other factors rarely relating to previous personal experience with Norway. An example illustrating this is Peter, a 39-year-old man from Germany, who grew up in East Germany, and at one point in his life renewed his contact to an old female friend, who had moved to Norway many years before:

… the lady … just wanted to say hello again after our more regular contact earlier. 15 years after the school time. …But there was too much to talk about, on telephone, so we decided that I should go to Norway to have the option of talking more. And this was very fateful.. the relationship bloomed again.. she showed me around (in Norway) … and I was impressed by what I saw … and a nature adventure (Peter, Germany, Study B)

Peter’s destination choice of Norway was an active choice at a time in his life when he felt ready to make the decision to migrate. He initially arrived in Norway on a temporary basis; then he became ‘captivated’ by Norwegian nature, and at the time we met him, he had decided to stay in Norway, four years after his choice of ‘destination’.

### From destination choices to LTC work

Some participants in our studies actively sought and secured LTC work as a means of entering their destination country. This was particularly true in the case of the UK, perhaps due to the strong role of UK LTC recruitment agencies across Europe (Cangiano et al. 2009; Jayaweera and Anderson 2008). From a migrant’s perspective, LTC work, while situated to some extent at the lower end of the labour market hierarchy, can still be considered a relatively attractive option. This is mainly because of the (un)availability of other jobs, especially for European migrants from recession-hit areas in East and South Europe who in many cases face barriers in terms of qualification recognition and language proficiency within the EU. Among EU participants in the survey part of Study A, 14 per cent indicated they joined the LTC sector because of ‘ease of securing a job in the sector’ compared to only 4 per cent among non-EU migrants. Berta, a 28-year-old Polish lady explained how she sought employment in the British LTC sector through an agency, while still in Poland, as a means of moving to the UK:

Yes [I already had the job before arriving], because I signed contract in Poland with agency and so, I had the accommodation and I just had the job, so I just actually came and the next day I went to work. I did not need a visa. Chop, chop decision, two days. It was really fantastic. (Berta, Poland, Study A)

In contrast, for some migrants, the decision to work in LTC was taken after arrival in both the UK and Norway. This can occur through a lens of pragmatism, although the aims and drivers were somewhat different in the UK and Norwegian cases. In the UK, LTC work was mainly seen as a way of securing ‘any’ work in the British labour market. In the Norwegian case, this was additionally considered as an opportunity to further develop their own qualifications, which in turn could improve future employability. Karolina from the UK study, a 24year-old Lithuanian lady, explained how she initially arrived to the UK seeking to work in ‘any’ job, then ended up ‘doing’ care work:

Basically, I didn’t want to work in my country. It was difficult here at the beginning, because I didn’t have any experience and stuff like that. It was hard to find any job, but I tried that kind of job [social care]. When I decided to do this kind of work, I [was] basically looking for all the different companies and I just walked in and she got me into it. She recruited me. Basically that’s it. (Karolina, Lithuania, Study A).

A similar observation of conscious access to a care job for further opportunities was observed in the Norwegian case, however not necessarily to facilitate the initial migration decision. Marija, who is 30 years old and from Lithuania too, exploring her pragmatic decision-making process said:

I did not find anything interesting to do, and there were language problems … everywhere …and I thought that I should keep working [as a care worker/personal assistant] and working, and I kept working and working and learning language …but because I was from abroad, I could only work 20 h a week… but when finishing the studies I started full-time working. (Marija, Lithuania, Study B).

Marija’s pragmatic consideration was to collect capital for her further plans of finding an interesting job based on her achieved qualifications.

### Crossing language and skills barriers

Many European migrants face barriers in accessing the skilled labour market (Benton et al. 2014), including qualification recognition and language proficiency. In the UK context, many European migrants, as compared to those from Commonwealth countries who grew up with English as their second language, arrived in the UK without these language skills. These differences in English language proficiency were emphasized by findings from the survey of Study A, where 15% of EU migrants indicated that they had some or major difficulties with working in the English language compared to only five per cent among non-EU migrants. However, for many migrants the choice of the UK as a destination was partially, and also pragmatically, made to develop language skills as an asset or ‘exportable’ capital for further ‘circular migration’ (Parreñas 2010). From a subjective rational perspective, this makes the UK an attractive country to choose as an ‘entry’ point, more than Norway. Anna, a 29-year-old Polish woman, explained how she considered learning the English language as an opportunity when choosing to come to the UK:

Poland was already in the union. That’s why it wasn’t a problem for me to be employed legal[ly] and that was the reason I came. … important was [that] I wanted to study English. I wanted to learn English in here. (Anna, Poland, Study A)

We found securing work in the LTC sector, in both the UK and Norway, offered many migrants the opportunity to overcome language proficiency barriers. Even the less accessible Norwegian language was not perceived as a barrier for many migrants to join the LTC sector even though some of them had difficulties in both the English and Norwegian languages. Peter explained how after arriving to Norway he became a personal assistant/care worker to earn money ‘*without really managing the language’*. However, while the English language is an ‘exportable capital’, the Norwegian language appeared to be an investment to enhance one’s opportunity while remaining in Norway as illustrated here by Leva. She is a Lithuanian 31-year-old woman who quickly made time and financial investments as part of a process of settling in Norway.

The day after I came to Norway I started a course in Norwegian language. And I then continued and continued … and I have thought that when invested so much, then I thought that Norway is a rich country. …where I might have the chance to get a new work … to find a job, and maybe if there are money, then there is new technology and everything is the best. (Leva, Lithuania, Study B)

In relation to challenges associated with skills and qualification recognition, we found that migrants adopt different strategies in the UK and Norway to overcome them. A common barrier was described by Adriana, a 43-year-old nurse from Romania, who ended up working in the Norwegian LTC sector.

I have sent all my documentation from the school [in Romania] to Oslo … but I got ‘not recognised’ on my education … because the school I went to is not registered in Norway … I need to do a six months’ course … but there is no course here [in the town she is living], only in Bergen and Oslo, so this is difficult for me having children at home [in Norway]. (Romania, Study B)

An important difference between Norway and the UK is the increase in higher education fees in the UK (except for Scotland; Dearden et al. 2012). Arguably, variable higher education tuition fees in Europe can drive higher numbers of international students away from the traditionally attractive UK destination to other European countries (Brooks and Waters 2011). Students in Norway are supported by the state through scholarships and low-rate loans, including non-Norwegian residents if they have held continuous full-time employment in the country, had a residence permit as an employee and paid tax during the previous year of study.^3^ Thus, being employed in a less-skilled job in the Norwegian LTC sector may open the door to other higher education qualifications for many migrants, particularly EU migrants who do not require a residence permit.

The UK, on the other hand, has a system for qualifications operating at a lower level: the Qualification and Credit Framework (QCF), with some elements (such as QCF Level 1) either offered to care workers without fees or supported by their employers as part of a training and qualification program (Department of Health [DH] 2009). This system gives migrants in the British LTC sector a chance to start a qualification process from a lower level when compared to Norway.

### Migrants’ rights and protection in different welfare and employment regimes

As many other welfare states in Europe, Norway and the UK developed their modern welfare states after WWII, but the development took off in quite different directions (Esping-Andersen 1999). Norway displays a distinct welfare model in comparison to that of the UK. More than other Nordic countries, Norway has ideals about universalism, social justice and redistribution of wealth (Brochmann and Hagelund 2010), including reducing migrant poverty (for non-EU migrants) (Hooijer and Picot 2015, p. 1890). Migrants, but particularly from within the EU, residing in Norway are intentionally given almost the same rights and duties as Norwegian citizens (Hatland 2011).

On the other hand, the UK welfare system is relatively more complex and much less universal than the Norwegian case, with the majority of social security systems being means-tested and having strict criteria. UK welfare rights are additionally stratified by and dependent on immigration status, forming a hierarchy of citizenship (Bolderson 2011). We found that knowledge of welfare rights, benefits and social security on offer formed an important part of migrants’ decision-process. Maria from the Czech Republic, 25 years old, compared the employment rights to maternity pay in her home country to that in the UK:

Back home, it doesn’t matter whether you work for the same company or for four different companies as long as you work for all this time, you are entitled to maternity leave. In here, it feels almost punishment. How dare you get pregnant right now. I’m missing my maternity pay, five weeks here. (Maria, Czech Republic, Study A).

The Norwegian employment and welfare protection system seemed more important for settling in rather than for choice of Norway as an initial destination. Migrants in Norway reflected more positively when comparing their entitlements in Norway in comparison to those in their home country. Helena from Romania compared social and health care in the two countries, concluding:

I really like Norway … and I don’t want to change the place. I started liking it when I started working … There are clear differences [between Romania and Norway]. Here it is much better. Yes, here people are taken better care of than in Romania … In Romania people have to get medicine on their own. (Helena, Romania, study B)

Another important aspect of empolyment rights relates to wages and protections offered through fair contracts and trade union membership. The UK-Norwegian differences in relaiton to the LTC sector were quite striking. When Elena, 27 years old, from Lithuania was asked whether she came to Norway for economic reasons, she had these reflections:

Yes, but I can’t say it is only for economic reasons, but primarily yes. … Now I work as an unqualified worker, but I earn five times as much as in Lithuania. Yes, and if I get a long shift, then I get as much as my brother is receiving for a whole month. Full time. … It is difficult for me to hear him saying ‘Yes, I get so much salary’. Then I’m thinking: ‘Oh shit’ I get so much … after just one day of work. (Elena, Lithuania, Study B)

Norway has avoided setting a National Minimum Wage (NMW) and wages are instead based on negotiations between representatives of the three labour market parties: employers, employees and local governments. LTC workers in Norway, including migrants, appear to have some influence over wages through these union negotiations. Despite this, the pay for LTC work is still relatively low in the Norwegian labour market (Gjertsen and Olsen 2012). However, from a migrant perspective, it would be the wage difference between the home country and the host country that contributes to their choices, as illustrated by Elena (see above).

In the UK, one major challenge of working in the LTC relates to the much lower wages than those in Norway. A significant minority of LTC workers in the UK are paid under the NMW with the majority paid on or just above the NMW (Hussein 2011; Gardiner and Hussein 2015; Hussein forthcoming). The British LTC workforce is also unregulated with low union density and currently facing increased presence of weak contractual protection and working in isolated environment with no formal support mechnisms (Rubery et al. 2015). Employers interviewed as part of study A acknowledged the fact that migrants, especially women, are likely to accept low pay in return for facilitating the act of migration itself.

I think women migrant workers will be channelled into private nursing homes, private old peoples’ homes, because they could be paid minimum wage, not going to make a lot of demands, work hard and work shifts, being available. (Human Resource Manager, Study A).

However, from a migrant’s perspective, calculating different options, it seems there is always a way around these difficulties. For example, many migrants developed various coping strategies to overcome issues of very low pay through working many hours, and avoiding renting costs by drawing on their own social networks or opting for live-in care work for accomodation purposes. Rolanda, a 40-year-old lady from Lithuania explains how social networks can be crucial in negotiating access to work and accomodation, even when there are clear language and financial barriers, and how such social capital enabled her to cope with such circumstances:

I had my friend, it [she] was my landlord after I move to different place. It’s one friend from Latvia, she helped me with this [getting job with the domiciliary care agency]. She say, if you like, I help you and I was not sure. Can I do this or not and it will be my [poor] English language all right in its place and I try. I made all application and then straight away to private place one time and then resident homes and then hospital and then mobility centres and I try everywhere. I was very happy everywhere. (Rolanda, Lithuania, Study A)

### The interplay of macro-social structures and their interpretation by migrants

**Fig. 3:**
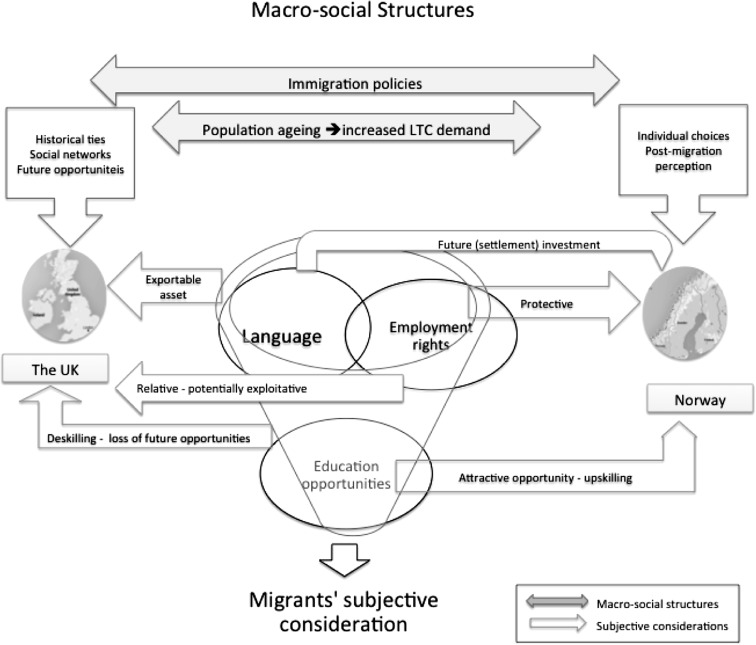
Summary of migrants’ subjective consideration of structural factors (and their potential impact) in relation to the UK versus Norway decision

Figure 3 summarizes the macro-social structures as perceived through a migrant’s perspective, using a framework of a migrant agency. The analysis indicates that the wider immigration policies act as facilitator for EU migrants to both countries; although from a migrant’s perspective and based on their social networks, the UK stands as a more desirable destination. Migrants consider Norway on a more individual basis and for specific reasons. The welfare regimes, while difference in the two countries, combined with ageing populations, offer opportunities for migrants to work formally in the LTC sector. Other factors including language, employment rights and educational opportunities are perceived and weighed differently for the two countries. The English language stands as an attractive exportable asset for many migrants, while the Norwegian language is perceived as an investment in the future particularly when combined with further educational opportunities. The British LTC sector offers very little employment protection and few qualification opportunities resulting in possible exploitation and de-skilling of migrants (Shutes and Chiatti 2012). On the other hand, the Norwegian LTC sector is perceived to offer employment protection and open opportunities for obtaining further qualifications and thus may act as means for up-skilling and further integration in the wider labour market.

## Discussion

The current analysis while offering important insights is limited in that it relies on a relatively small number of cases of migrants. The findings presented here are based predominantly on qualitative analysis of interviews, which provided a very differentiated picture of the major considerations involved in migrant’s decisions related to long-term care work in the UK and Norway. Other researchers may wish to examine some of these findings through developing larger quantitative studies. The latter would help to extend the current evidence based on the aforementioned issues and on possible conditions and consequences of these subjective considerations.

The analysis presented here offers two important contributions to the literature on ageing, migration and care. One is related to the understanding of migrant care workers as active agents when confronting immigration policies, labour market regulations, qualification systems and language barriers. Thus macro-social structures are reflected upon differently from an individual micro level perspective when migrants consider their country of destination. The analysis also indicates that current European welfare regimes are more complex than has been identified by Esping-Andersen’s (1999) model. This relates in particular to the role of marketization policies reshaping the care market in Norway and the UK (Meagher and Szebehely 2013) in the direction of facilitating the inclusion of more migrants in the care market. This means that both liberal (UK) and social democratic (Norway) care models imply higher demand for migrant care workers in their formal labour market, unlike the classification provided by Van Hooren (2012).

A rational choice perspective, as used here, is inherently multi-level in structure and takes into account the individual’s circumstances, their environment and the social structure (Coleman 1990). Within this model, the institutional structure, in this case the immigration and wider welfare policies of the countries, acts as context for the individual decisions made. In the case of LTC, migrants use the institutional context of EU-free labour mobility to consider the act of migration and use their social networks and the social structures as means of facilitating this action. The welfare regime of a country offers another dimension in considering the opportunities and losses of joining a low paying sector such as LTC. Migrants in our study showed clear understanding of different options and the opportunities each country could offer. In the case of the UK, learning the exportable English language is perceived as an important outcome for future options and choices even if it is associated with little employment protection and rights. On the other hand, the Norwegian generous welfare and employment regime is perceived as making it worth investing in a less transferable language skill, especially with the option of low-cost higher education opportunities. Within this context, we find individual judgment to be as important as the structural institutional factors (such as the welfare regime or employment rights and protection in a country) for migrants’ destination and work decision-processes. Thus, the actions of individual migrants are shaped by both individual and structural factors and explained by long-term motivations to achieve certain outcomes, consistent with Max Weber’s ‘Thick’ model of rational choice (Hechter and Kanasawa 1997).

The EU political map is currently very fluid with more countries applying to join, while the UK has recently voted to leave the EU (Brexit). This was preceded by recent changes in British public opinion and attitude towards migrants, which has been increasingly negative since early 2000 and particularly since 2008 (Ford et al. 2015). The impact of Brexit is not yet clear and will surely influence the subjective decision-making process of EU migrants and see countries like Norway weighing up as a more attractive destination for European LTC migrant workers.

## Conclusion

Using the two cases of the UK and Norway, we have showed that Western European countries operating different care models increasingly rely on migrant workers particularly from Eastern Europe to meet the escalating demands of formal LTC sectors. Within the context of free European labour mobility, the decisions of Eastern European workers to migrate and work in LTC sectors are affected by a set of structural and subjective factors relating to macro and micro levels. While immigration legislations and policies are key factors regulating immigration at the macro level, other structural factors such as the type and implications of the welfare regime, the employment conditions related to labour market characteristics as well as language and qualification recognition are factors that are subject to interpretation by migrants themselves. Negotiating these factors depends on the way they are approached, interpreted and managed by migrants. Within such a process, the migrant’s perspective, including individual choices based on considerations, reflects how individuals actively negotiate structural conditions and barriers to achieve the next step in their migratory journey.

## Footnotes

1 Defining LTC as human health and social work acticities (SIC- 86–88)

2 The A8 countries are a group of eight of the 10 countries that joined the European Union during its 2004 enlargement. They are commonly grouped together separately from the other two states that joined in 2004, Cyprus and Malta, because of their relatively lower per capita income levels in comparison to the EU average. These are: Czech Republic, Estonia, Hungary, Latvia, Lithuania, Poland, Slovakia, Slovenia.

3 See: https://www.lanekassen.no/nb-NO/Toppmeny/Languages/Financial-support-for-foreign-students/Who-is-eligible/
